# Neuroscience research in Africa: Current status

**DOI:** 10.1016/j.ensci.2015.10.005

**Published:** 2015-11-10

**Authors:** Foad Abd-Allah, Najib Kissani, Anthony William, Mohammed Ibrahim Oraby, Ramez Reda Moustafa, Ehab Shaker, Mohamed Soliman El-Tamawy, Raad Shakir

**Affiliations:** a Department of Neurology, Cairo University, Cairo, Egypt; b Department of Neurology, Marrakech University Hospital, Marrakech, Morocco; c University of Ghana Medical School, Neurophysiology Unit, Ghana; d Department of Neurology, Beni-Suef University, Beni-Suef, Egypt; e Department of Neurology, Ain Shams University, Cairo, Egypt; f Department of Neurology, Imperial College, Charing Cross Hospital, London, United Kingdom

**Keywords:** Africa, Neuroscience, Publications

## Abstract

There are limited data on the contribution of the African continent to neuroscience research and publications. This review aims to provide a clear view on the state of neuroscience research among African countries, and to compare neuroscience research within the 52 African countries. A literature review search was conducted for all published articles by African authors in both local and international journals using Medline and other primary databases. Neuroscience represents 9.1% of the total medical publications. The highest percentage of neuroscience publications comes from South Africa. There is a positive correlation between the Gross Domestic Product and the total number of neuroscience publications among African countries. There is therefore an urgent need to develop strategies to improve neuroscience research in African countries.

## Highlights

• This is a review article covering the African neuroscience publications during the period from Jan. 2003 to Jan. 2013.
• South Africa occupied the first place among the top ten publishing African countries followed by Egypt.
• Most of the published articles are in the field of clinical neurology rather than basic neuroscience.
• The intensity of publications correlating with GDP, not however with the number of medical schools

## 1 Introduction

There have been tremendous advancements in the field of neurosciences over the last few decades with contributions from neuroscientists from across the world. This has been diverse and varied. The African continent, despite all of its shortcomings is striving to catch up with the more scientifically advanced world in the domain of research and publications.

There were a huge effort from different organizational bodies that have been promoting and sponsoring neuroscience research in Africa, e.g. the Pan African Association of Neurological Sciences (PAANS), the International Brain Research Organization (IBRO), the European Academy of Neurology (EAN), the National Institutes of Health (NIH), the NIH Medical Education Partnership Initiative (MEPI), the Wellcome Trust, the Wellcome Trust DELTAS Africa program, African chapters of Neuroscience society and the World Federation of Neurology (WFN).

The aim of this review is to address the current status of neuroscience research in Africa, to ascertain the contributions of African academics to the development of neuroscience and to perhaps offer some recommendations to bolster the level of research activities among different countries in the continent.

## 2 Literature search and data collection

We searched the total scientific medical publications, neuroscience research publications (basic and clinical) and the language of publications in 52 recognized African countries over a span of 10 years from January 2003 to of January 2013.

We performed an initial Medline search using the Medical Subject Heading (MeSH) term “neuroscience” and a series of terms ensuring to capture all neuroscience publications. We deliberately chose the comprehensive MeSH term “neuroscience” and “nervous system” to cover all potentially relevant studies and publications. English language and non-English language articles were included from 1st of January 2003 to 1st of January 2013. After the initial Medline search was validated, the search was expanded to other primary databases like EMBASE in order to retrieve abstracts, which were not identified on Medline. In addition, papers were found by a manual search using references cited in original study papers and reviews. The first stage of inclusion was based on screening titles and abstracts, specifically, those including “neuroscience”, “brain”, “nervous system”, “neuron”, “cerebral”, “neurogenetics”, ”neurodegenerative”, “neuroinfection”, “stroke” and “epilepsy”. The final list of abstracts was prepared for review by downloading search results from each database to Reference Manager 12 (Thomson Reuters, New York City, New York). We tried to streamline the results in order to eliminate repetition and duplications. For the total medical publications for each country, we used the name of the country and medical scientific publications, and then we proceeded by adding the number of generated publications for each country giving us the total number of publication for Africa for the past 10 years.

## 3 Statistical methods

Data were entered and analyzed using IBM SPSS Version 20. Number and percent were used to describe qualitative variables. Spearman rho bivariate correlation was used to test the relation between quantitative variables. P value less than or equal to 0.05 was considered to be significant.

## 4 Results

The total number of neuroscience publications was 12,590 out of 137,714 total medical publications, which accounts for only 9.1% of the total medical publications and most of those publications were not in international/indexed journals or databases, but rather in local journals.

Most neuroscience publications were in the English language (76.2%). The most common field of neuroscience publications was clinical neurology (70.9%).

Out of 52 countries, the top 10 countries (contributing 80.6% of the total neuroscience publications) were selected to demonstrate the most active publication areas, the language of publications and to assess factors which may have an effect on neuroscience research such as Gross Domestic Product (GDP) and the number of medical schools in such countries (Table 1, Table 2).

**Table 1:** The top ten African countries in neuroscience publications.

	Total medical publications	Neuroscience publications			Common field		Language of publications
		Number	Percentage to total African neuroscience publication	Percentage to total medical publications	Type	Percentage	
South Africa	31,235	3415	27.12%	10.93%	Clinical	74%	English
Egypt	17,112	2478	19.68%	14.48%	Clinical	66.14%	English
Nigeria	13,532	1079	8.57%	7.97%	Clinical	76%	English
Tunisia	7329	775	6.15%	10.57%	Clinical	84.26%	English/French
Congo	3813	762	6.05%	23.57%	Clinical	58.53%	English
Morocco	3933	587	4.66%	7.84%	Clinical	74.11%	English
Mali	1925	351	2.78%	9.3%	Clinical	97.7%	English
Kenya	6473	289	2.29%	19.98%	Clinical	85.12%	English
Sudan	2693	211	1.67%	3.24%	Clinical	97.6%	English
Togo	857	202	1.6%	7.3%	Clinical	94.7%	English

**Table 2:** Number of population, number of medical schools and per capita income for the top ten publishing countries in Africa.

	Number of populations (millions)	Number of medical schools	GDP per capita (current US$)
South Africa	51.1	10	7507.67494
Egypt	82.3	19	3187.312591
Nigeria	170.1	26	1555.410979
Tunisia	10.8	4	4236.793631
Congo	4.2	1	3153.739461
Morocco	32.6	4	2902.329958
Mali	16	1	693.9843612
Kenya	43	2	864.7447928
Sudan	33.5	26	1580.004017
Togo	6	1	574.1195223

The highest rate of neuroscience publications was reported in South Africa, 3415 publications (27.12% of the total neuroscience publications), 74% of them were clinical research articles.

**Fig. 1:**
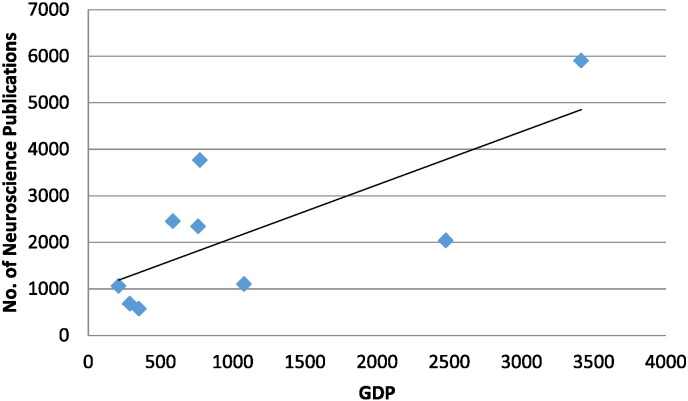
The relation between GDP and the number of neuroscience publications.

There was a positive correlation between the Gross Domestic Product (GDP) and the total number of neuroscience publications (Fig. 1).

**Fig. 2:**
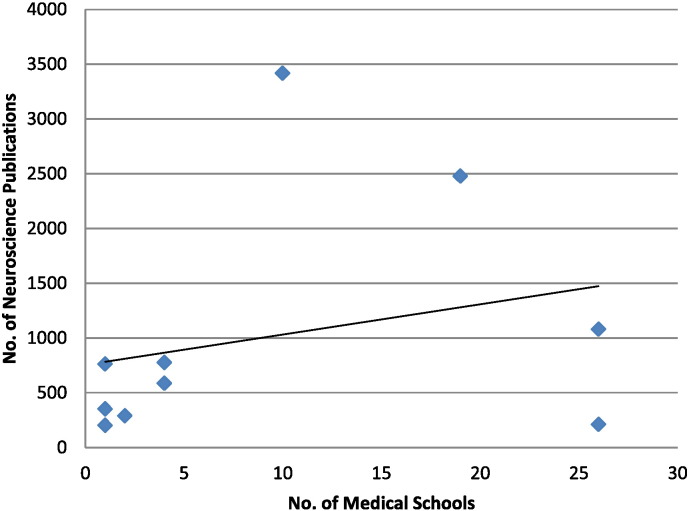
Correlation between number of medical schools and the neuroscience research publications.

There was no statistically significant association between number of medical schools in each country and the neuroscience research publications (Fig. 2).

Strong positive correlation was found between GDP and Number of Neuroscience Publications r = 0.667, p = 0.05 using Spearman rho bivariate correlation analysis (variables were not normally distributed), Linear fit line was added R^2^ = 0.523.

Number of medical schools was not correlated with Number of Neuroscience Publications r = 0.27, p = 0.482. Linear fit line was added R^2^ = 0.07.

## 5 Discussion

Neuroscience is one of the fastest growing areas of scientific interest and investment in its research and education is likely to be an important driver of scientific growth and social development [2], [12].

The current study addresses the contribution of African countries to global neuroscience research in the continent and its correlates. The main output of neuroscience research was much more slanted towards clinical research rather than to basic science.

Many challenges are facing scientists in African countries such as inadequate curricula to prepare students to enter neuroscience as a career choice [5], a pervasive lack of funding, inadequate research infrastructure and career development programs, small number of neuroscientists in the African continent.

In contrast to resource limited African countries, developed countries with availability of scientific grants, as well as a well established research systems and laboratories make the comparison difficult. As a result, most young and upcoming scientists from Africa do not take up neuroscience as a career choice, and when they do, they tend to immigrate to developed countries, often not to return [6].

Access to technological tools, information access and other equipment and supplies to assist research work is not always possible [10]. However, a further difficulty facing African researchers is dissemination of findings from their research to other parts of the world [10]. Most of the information published in African journals is largely not included in major databases [9].

The highest rate of neuroscience publications was reported from South Africa. In that country, there is an increasing realization of the need to promote indigenous health research [7]. Since 1994, considerable policy-level steps were taken to reorient health research towards the needs of the vast majority of the population. These steps led to the publication of the Health Research Policy of 2001, whose aim was to develop a national health research system that contributes to equity in health development [7]. In 2003, the government developed a new funding mechanism, which entailed funding tertiary institutions based on their research outputs (i.e. number of publications and number of postgraduate students produced). A national survey of research and development in 2008/2009 recorded gross domestic expenditure on research and development of 1.5 billion Dollars, representing a 13% nominal increase over that of 2007/2008 [7].

Egypt, contributing 19.7% of neuroscience publications of Africa, is the most populous country in Middle East and second most populous in Africa after Nigeria. Egypt has a relatively advanced educational system with about 19 academic institutes [8], a well established local post graduate programs, and many post-doctoral exchange programs and fellowships established with western countries. In September 1999, there was a presidential declaration urging all parties in the country to accord top priority to research activities and to use new technologies. Health research is being given a firm commitment at the highest political levels.

Importantly, on our study, GDP showed a significant positive correlation with the number of neuroscience research publications. This may explain the wide variability in number of neuroscience research publications between African countries, as there is a wide difference between them in the national gross domestic product measured by GDP. The findings corroborate those of previous bibliometric analyses which demonstrated that health research productivity worldwide is largely dependent on each country's GDP [1], [3], [4], [10].

There was, however, no association between the number of medical schools in each country and the number of neuroscience research publications. This finding may indicate that medical schools were perhaps concentrating on undergraduate education for obvious reasons. This left their ability to use available research evidence to guide policy, develop programs, strengthen practice and maximize the use of resources in order to improve the research environment, lagging behind. This should be as important a goal rather than increasing the numbers of medical schools without clear, well developed undergraduate and postgraduate programs which encourage research.

A recent finding indicating that the most important factors for improving the value of health research in the WHO African Region are physician density, total health expenditure, private health expenditure, research and development expenditure, human development index and the number of journals [11].

Short term and long term strategies should be implemented to improve neuroscience research in African countries, but in the meantime it is reasonable for African countries to implement the World Health Organization (WHO) Strategy on Research for Health, which include: strengthening of the research culture (organization); focusing research globally on priority health needs (priorities); helping to strengthen national systems for health research (capacity); promoting good practice in research (standards); and strengthening links between health research and health (translation) [11].

This study presents an interesting data on the state of research in the neuroscience in Africa, yet it has some limitations. For example, it is not clear; whether the source of funding for some published studies came from African or non-African countries.

The African continent has the lowest ratio of number of Neurologists/population ranging from 1 Neurologist for 600,000 to 7 million with discrepancies between sub-Regions.

Recently, the African Academy of Neurology (AFAN) is now established following a historical meeting in Dakar, August 2015. This will allow African neurologists to meet on a large scale and with all sub-regions to discuss the present and future of neurology in Africa and to build collaborations between them. One of the main goals of AFAN is promoting neuroscience research across the continent.

## 6 Conclusion

This study presents an interesting ‘snapshot’ of the state of research in the neurosciences in Africa. The current data bring a valuable insight into the African contributions to research in neurosciences. The proportion between the African countries and the finding of its intensity correlating with GDP, not however with the number of medical schools are new and useful. Another future article could compare the GDP per capita, coupled with no. of publications of African countries with the same coupled GDP/capita and publications of the well developed world.

## Disclosure

The authors report no conflicts of interest.
